# Case Report: Right atrial organized thrombus three years after tricuspid annuloplasty

**DOI:** 10.12688/f1000research.129157.1

**Published:** 2023-01-03

**Authors:** Mohannad Abbass, Silvia Mariani, Sami Musa, Nicoletta Erba, Franco Masini, Salvatore Lentini

**Affiliations:** 1 Salam Centre for Cardiac Surgery, EMERCENCY ONG ONLUS, Khartoum, Sudan

**Keywords:** tricuspid valve repair, right atrial thrombus, intracardiac mass, follow-up

## Abstract

**Background:** Occurrence of right atrial masses, especially in patients with history of cardiac surgery, is rare. Differential diagnosis between malignant and non-malignant aetiologies might be cumbersome, and surgery is often required to prevent complications or disease evolution.

**Case:** We report the case of a 16-year-old girl from a rural area of Sudan, who underwent surgery for a modified De Vega’s tricuspid annuloplasty, and mitral and aortic valve replacement with mechanical prostheses. The patient was on regular follow-up but demonstrated a poor compliance to anticoagulation therapy with a time in therapeutic range between 52% and 20%. She remained asymptomatic, but a right atrial mass was diagnosed by transthoracic echocardiography during a follow-up visit 41 months after the first operation. Surgical removal of the mass revealed an organized thrombus arising from the point where the Prolene stitches for the tricuspid annuloplasty were previously passed. The patient recovered from surgery, was discharged home on post-operative day 10 and the first follow-up visit at 30 days after discharge confirmed a good clinical status and a normal transthoracic echocardiography (TTE).

**Conclusions:** This case report describes the diagnostic and therapeutic work-out of a thrombus formation on the suture lines of a tricuspid annuloplasty. Moreover, it highlights the importance of a strict and long follow-up after valvular surgery and of the adherence to anticoagulation therapy, especially for patients living in rural areas of developing countries.

## Introduction

Intracardiac masses are not frequent and they may arise in all of the four chambers of the heart. ^1^ Their aetiologies include thrombi, vegetations, and neoplasms. ^1^ Despite the pivotal role of echocardiography and other imaging techniques, differential diagnosis might be challenging, and surgery might be indicated to avoid complications and rule out malignancies. Intracardiac masses may arise also in patients that underwent previous cardiac surgery with implantation of valve prosthesis or valve repair. ^2^^,^^3^ Herein, we report a case of a girl that underwent triple valve surgery and was diagnosed with a right atrial mass more than three years after the indexed operation. This case report follows the CARE guidelines. ^12^

## Case presentation

We present the case of a 16-year-old Black African girl from a rural area of Sudan, with a history of recurrent tonsillitis, chest infections and untreated rheumatic fever. She was first referred to our hospital due to severe mitral, aortic and tricuspid regurgitation when she was 13 years old. Indication for urgent surgery was confirmed. The patient underwent mitral valve replacement with a 27 mm SJM Master mechanical prosthesis (Abbott, Burlington, MA USA), aortic valve replacement with a 19 mm SJM Regent mechanical prothesis (Abbott, Burlington, MA USA), and tricuspid annuloplasty with two separate Prolene 4/0 sutures with pledgets. The postoperative period was uneventful, and the patient was discharged home in good general condition on post-operative day 10. The pre-discharge transthoracic echocardiography (TTE) showed a mildly depressed left ventricular systolic function (46%), good result of the tricuspid repair with mild residual regurgitation and good function of the mechanical prostheses. The patient remained in regular follow-up at our outpatient clinic, restarted a normal life and went back to school. Two years after surgery she experienced menorrhagia with severe anaemia (haemoglobin: 5.6 g/dl) and oral aspirin (100 mg/day) was discontinued. She demonstrated a low compliance with the anticoagulation therapy (warfarin with a target international normalized ratio of 2.5–3.5), and a time in therapeutic range (TTR) of 52% during the first year after surgery, 34% during the second year, 34% during the third year and 20% during the first six months of the fourth year. No history of hypercoagulopathy and contraceptive use was reported. The patient remained in sinus rhythm during the whole follow-up.

**Figure 1.**
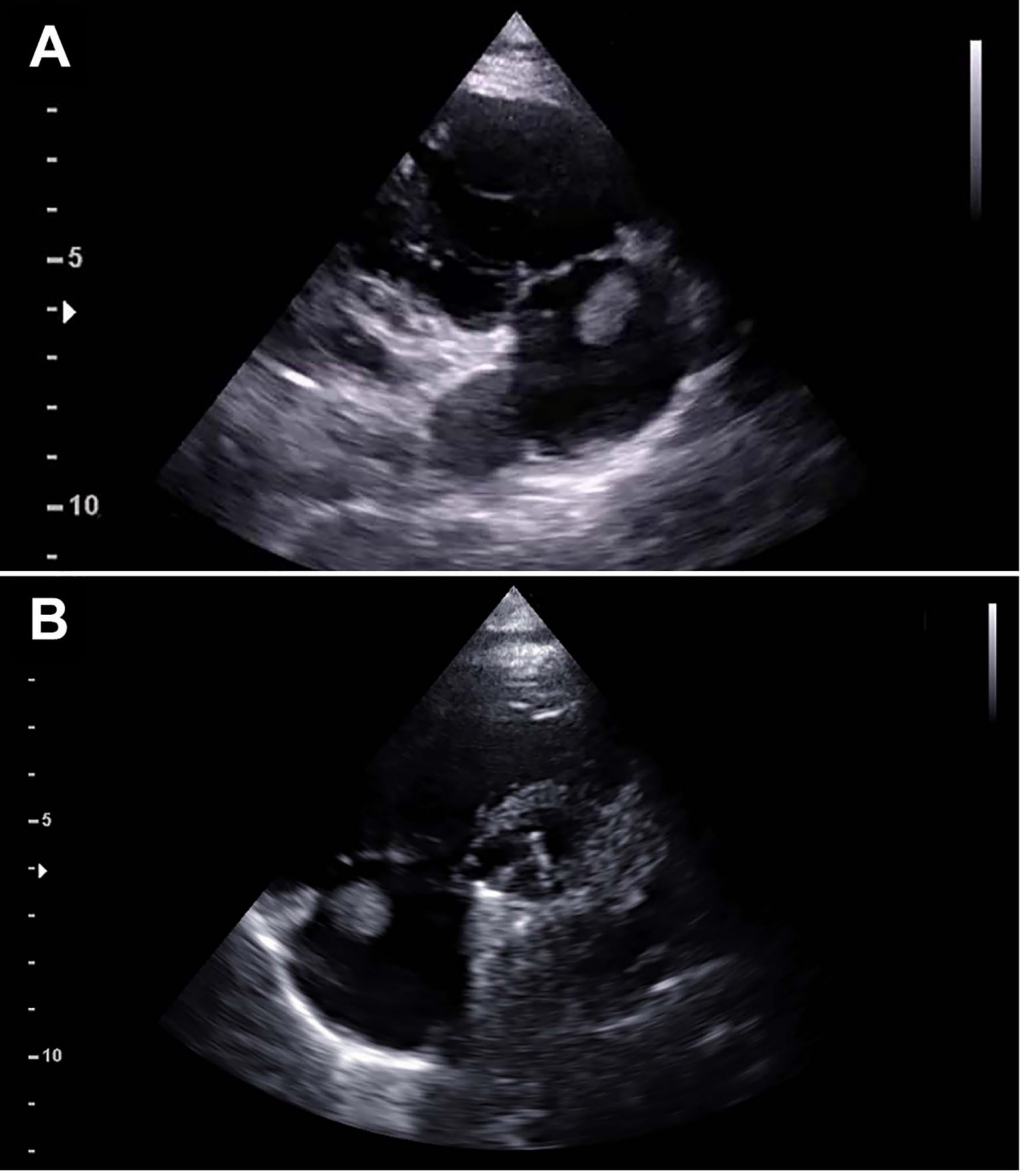
Preoperative echocardiography. Preoperative echocardiography demonstrating a mass measuring 11×12 mm attached at the junction of the right atrium and tricuspid annulus as shown in the right ventricle inflow view (A) and parasternal short axis view (B).

At the follow-up visit 41 months after surgery, the TTE showed a mobile mass measuring 10 x 15 mm and arising from the atrial wall just above the tricuspid annulus ( Figure 1). The right atrium was normal in size and structure (diameter: 31 mm; area: 15 cm ^2^). The mass appeared mobile with the cardiac cycle, but no interference with the tricuspid valve function was noticed. Surgical indication was given based on the significant dimension of the mass, its position close to the tricuspid valve, the risk for embolization and the possible neoplastic nature.

**Figure 2.**
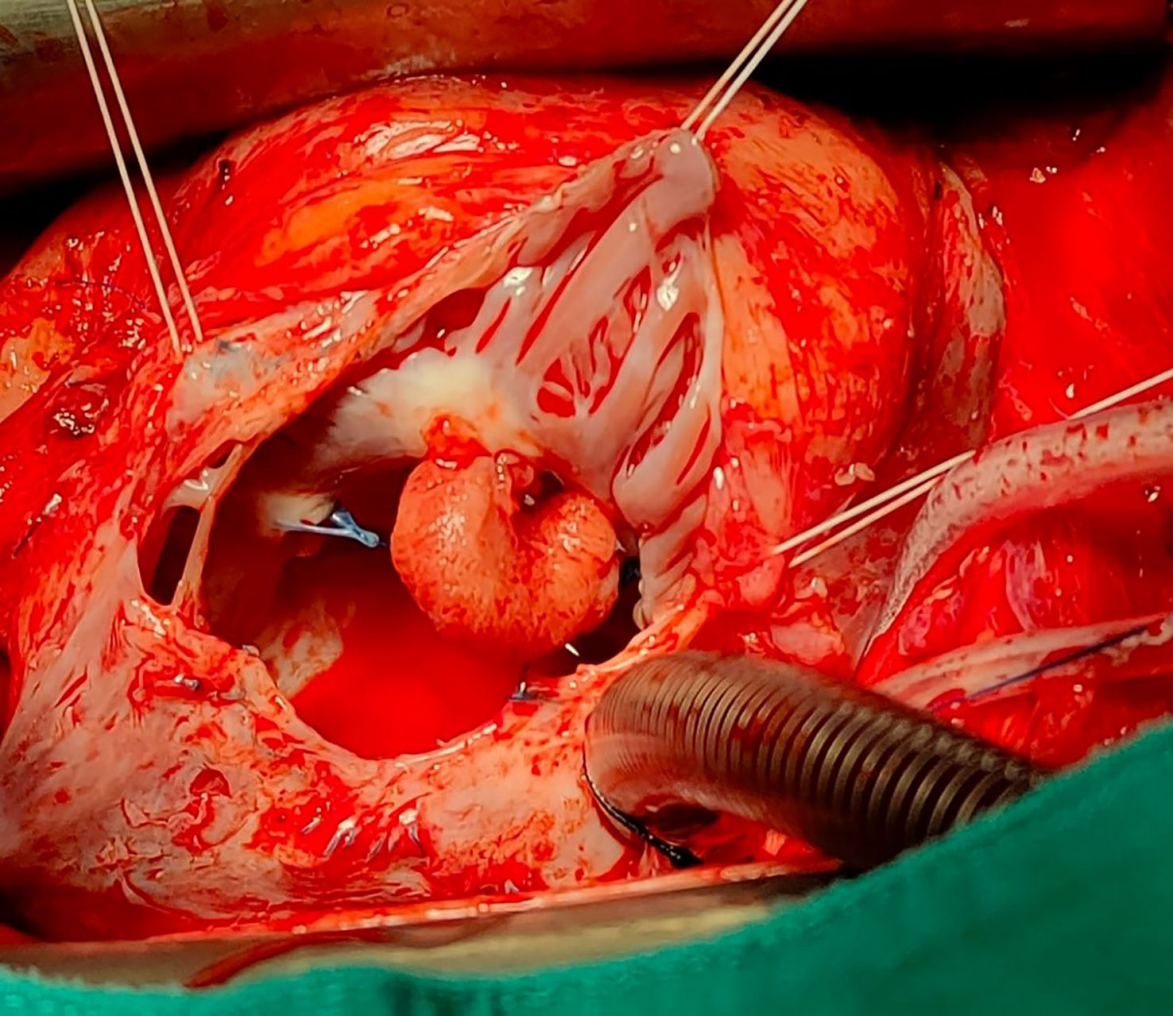
Intraoperative findings. Intraoperative surgical view demonstrating the presence of a pedicled mass attached to the atrial wall between the pectinate muscles and the anterior tricuspid annulus.

The patient underwent re-sternotomy, cardiopulmonary by-pass was established with bicaval and aortic cannulation, the heart was arrested, and the right atrium was opened. The mass appeared to be attached to the atrial wall between the pectinate muscles and the anterior tricuspid annulus ( Figure 2). In detail, the mass was arising from the point where the Prolene stitches for the tricuspid annuloplasty were previously passed but did not involve the valve leaflets. The mass had a curved shape and hard texture with a smooth surface. It was removed from the atrial wall using a knife, the atrium was closed, and the surgery completed. The macroscopic analysis showed a 15 × 15 × 8 mm homogenous light brown soft-tissue mass. The microscopic section showed several layers of hyalinized tissue containing red blood cells, scattered mixed inflammatory cells and fibrosis. The histological features were consistent with an organized thrombus.

The post-operative course was uneventful, and the patient was discharged home 12 days after surgery. The first follow-up visit at 30 days after discharge confirmed a good recovery and a normal TTE.

## Discussion

Occurrence of right atrial masses, especially in patients with history of cardiac surgery, is rare. This case report describes the diagnostic and therapeutic work-out of a thrombus formation on the tricuspid valve annulus after a modified De Vega’s annuloplasty. Moreover, it highlights the importance of a strict follow-up after valvular surgery and of the adherence to anticoagulation therapy, especially for patients living in rural areas of developing countries. To the best of our knowledge, this is the first report of a long-standing thrombus developed on the suture lines of a tricuspid annuloplasty and diagnosed more than three years after surgery.

Many types of masses can be found in the right atrium, from benign thrombus to malignant sarcoma. ^1^ For example, angiosarcoma may infiltrate the right atrioventricular junction, the atrial wall and also the tricuspid valve. ^4^^,^^5^ Lymphoma, hamartoma and pericardial mesothelioma can also affect the right atrium. ^6^^,^^7^ Vegetations on the tricuspid valve can be seen in case of infective endocarditis while non-infective masses have been reported in patients with antiphospholipid antibody syndrome. ^8^ Calcified masses such as the calcified amorphous tumour have been reported in patients with end stage renal failure, ^9^ and cardiac hydatid cysts on the tricuspid valve has also been described. ^10^ In case of previous cardiac surgery, the presence of prosthetic material can orient the aetiological diagnosis toward the presence of a thrombus. ^2^^,^^3^ Nevertheless, all other causes cannot be excluded, especially when the mass presents years after the operation.

Diagnosis is usually made initially by imaging through TTE as first-line modality, and cardiac computed tomography or magnetic resonance to better characterize the tissues. Finally, 18-Flurodeoxyglucose positron emission tomography may identify an increased metabolic activity of tumours. In the case described above, echocardiography was the only diagnostic tool available and precise differentiation among all aetiologies was not possible. However, yearly follow-up visits and TTE were pivotal to diagnose the mass and give surgical indication before the occurrence of any complication.

Finally, direct excision of the mass and subsequent histological analysis revealed the thrombotic origin of the mass. Although right-sided prosthetic materials have demonstrated a higher thrombotic potential than their left-sided ones, there are no specific postoperative antithrombotic management recommendations after tricuspid valve repair, especially in the case of all types of De Vega’s annuloplasty techniques. ^3^ Moreover, the association between tricuspid procedures and other indications for postoperative anticoagulation ( *e.g.*, concomitant left-sided mechanical valves like in the case presented) may contribute to the low incidence of this diagnosis, especially in case of infrequent TTE and follow-up contacts with the patient. This may become a problem in rural areas or developing countries where the follow-up programs might be difficult. ^11^ Moreover, difficult access to the tertiary cardiac surgery centre can reduce the patient’s compliance to the anticoagulation therapy as it happened in the presented case. Indeed, she demonstrated a low TTR with a decreasing trend as time passed since the first surgery. We can, thus, speculate that such a low compliance could have favoured the development of a thrombus over the annuloplasty stitches. Further screenings for any hypercoagulopathy could have helped in the characterization of the case but they could not be performed for economical and logistic reasons.

In conclusion, this case report highlights the importance of adequate follow-up programs for young patients undergoing valve surgery for rheumatic heart disease in developing countries. Moreover, it shows how tricuspid annuloplasty can carry a certain degree of thrombotic risk, even years after surgery. Further studies are required to investigate the fate of patients receiving tricuspid annuloplasty associated to left-sided valve surgery for rheumatic heart disease.

## Consent

Written informed consent for data collection and publication was obtained from the patient and the patient’s mother, as legal guardian. We confirm that we have obtained permission to use images included in this presentation from the patient and the mother, as legal guardian.

## Data Availability

All data underlying the results are available as part of the article and no additional source data are required. Figshare: CARE checklist for ‘Case Report: Right atrial organized thrombus three years after tricuspid annuloplasty’. https://doi.org/10.6084/m9.figshare.21640205. ^12^ Data are available under the terms of the Creative Commons Zero “No rights reserved” data waiver (CC0 1.0 Public domain dedication).
