# Supplementary material for: Case Report: Right atrial organized thrombus three years after tricuspid annuloplasty
Source: F1000Res. 2023 Jun 1;12:6. Originally published 2023 Jan 3. [Version 2] doi: 10.12688/f1000research.129157.2 (PMC10285360; doi:10.12688/f1000research.129157.2)
Supplement: Peer review history

# Peer review: Reviewer response for version 2

**DOI:** 10.5256/f1000research.148424.r176264

**Published:** 2023-06-08

**Authors:** Sossio Perrotta

**Affiliations:** 1 Department of Molecular and Clinical Medicine, Sahlgrenska Academy, Gothenburg University, Gothenburg, Sweden

I think that the answers provided by the author are exhaustive and that the manuscript can be approved for indexing.

Are enough details provided of any physical examination and diagnostic tests, treatment given and outcomes?

Yes

Is the case presented with sufficient detail to be useful for other practitioners?

Yes

Is sufficient discussion included of the importance of the findings and their relevance to future understanding of disease processes, diagnosis or treatment?

Yes

Is the background of the case’s history and progression described in sufficient detail?

Yes

Reviewer Expertise:

NA

I confirm that I have read this submission and believe that I have an appropriate level of expertise to confirm that it is of an acceptable scientific standard.

# Peer review: Reviewer response for version 1

**DOI:** 10.5256/f1000research.141821.r158882

**Published:** 2023-05-03

**Authors:** Sossio Perrotta

**Affiliations:** 1 Department of Molecular and Clinical Medicine, Sahlgrenska Academy, Gothenburg University, Gothenburg, Sweden

I would like to gratulate the author for reporting this interesting and rare case of a patient with a thrombus formation on the tricuspid valve annulus after a modified De Vega’s annuloplasty. The patient underwent surgery with removal of the thrombus and discharged home 12 days after surgery.

 The article is well written, but few points should be clarified. I would like the authors to comment on the following points

 The author should provide some information about the preoperative symptoms and the preoperative echocardiographic finds at the time of the first operation.

 Which was the anticoagulation therapy at discharge?

 The author states that aspirin was discontinued  two years after the surgery. Why was aspirin needed two years after the surgery? Probably due to the low compliance to anticoagulation therapy. But since the compliance to anticoagulation was low at the third and fourth year was the aspirin reintroduced?

 I notice that the author uses two specific mechanic valves in aortic and mitral position. On the market is available a different mechanical valve prosthesis, that can be used in both mitral and aortic position. For this specific mechanical valve the anticoagulation therapy can be carried out with a lower INR in combination with aspirin.  Has the author thought to change the used mechanical valves in consideration to the special social contest in which he practices and the difficulties for the patients to adhere to the needed anticoagulation therapy?

 The author states that the patient had a low compliance with regard to the anticoagulation therapy, but no information is given if the patient had trombi on the mitral and aortic valves or if she had peripheral embolism.

 To remove the mass in the right atrium, the patient underwent a second surgery and the heart was arrested. Why the author used this surgical approach? This kind of surgery can be performed as well with a beating heart. Since the patient had a mildly depressed left ventricular systolic function (46%), why to expose this heart to ischaemic time when it was possible to avoid it?  

 I think the the article can be deemed suitable for indexing with minor corrections

Are enough details provided of any physical examination and diagnostic tests, treatment given and outcomes?

Yes

Is the case presented with sufficient detail to be useful for other practitioners?

Yes

Is sufficient discussion included of the importance of the findings and their relevance to future understanding of disease processes, diagnosis or treatment?

Yes

Is the background of the case’s history and progression described in sufficient detail?

Yes

Reviewer Expertise:

Endocarditis

I confirm that I have read this submission and believe that I have an appropriate level of expertise to confirm that it is of an acceptable scientific standard, however I have significant reservations, as outlined above.

# Response

**Published:** 2023-05-30

**Authors:** Salvatore Lentini

**Affiliations:** The Salam Centre for Cardiac Surgery, Sudan

We are grateful for the opportunity to submit a revised version of our manuscript ******“****** ******Case Report: Right atrial organized thrombus three years after tricuspid annuloplasty****** ******”****** to *F1000 Research* and are thankful for the very constructive and helpful comments of the Reviewers. In addition to responding to the comments, we revised the manuscript, and hope that the changes and responses satisfy the Editors and Reviewers` requests.

****Reviewer 2****

**I would like to gratulate the author for reporting this interesting and rare case of a patient with a thrombus formation on the tricuspid valve annulus after a modified De Vega’s annuloplasty. The patient underwent surgery with removal of the thrombus and discharged home 12 days after surgery. The article is well written, but few points should be clarified. I would like the authors to comment on the following points.**

 We are grateful to the Reviewer for the very constructive and helpful comments. In addition to responding to the comments, we revised the manuscript, and hope that the changes and responses satisfy the Reviewers` requests.

**The author should provide some information about the preoperative symptoms and the preoperative echocardiographic finds at the time of the first operation.**

 We thank the Reviewer for this suggestion. We added a brief description of the pre-operative symptoms and echocardiographic findings.

**Changes: case presentation**

 Her symptoms started with shortness of breath on exertion, dry cough, palpitations and lower limb oedema. She was first referred to our hospital due to signs of acute decompensation (raised jugular venous pressure, limb oedema and dyspnoea) when she was 13 years old (body mass index 12.65). Transthoracic echocardiography showed retracted rheumatic mitral and aortic leaflets associated with severe mitral and aortic regurgitation. The left atrium and ventricle were dilated, and a secondary severe tricuspid regurgitation was diagnosed.

**Which was the anticoagulation therapy at discharge?**

 The Reviewer pointed out an interesting clinical detail. We modified the manuscript adding details about the anticoagulation therapy at discharge:

**Changes: case presentation**

 The anticoagulation therapy included warfarin with a target international normalized ratio of 2.5–3.5 and was associated with oral aspirin (100 mg/day).

**The author states that aspirin was discontinued  two years after the surgery. Why was aspirin needed two years after the surgery? Probably due to the low compliance to anticoagulation therapy. But since the compliance to anticoagulation was low at the third and fourth year was the aspirin reintroduced?**

 We thank the reviewer for this interesting question. Aspirin was associated with warfarin as the patient was a woman in fertile age living in a rural area. Thus, the patient was at higher risk of low compliance for the anticoagulation therapy and a possible pregnancy would have required association with anti-platelet therapy as previously reported in a study from our group on pregnancy outcomes in women with mechanical valve prostheses using vitamin K antagonist therapy (PMID: 35899137), and following guidelines (PMID: 33342587). After the menorrhagia experienced by the patient, aspirin was not re-introduced despite the low compliance due to the previous bleeding event.

**Changes: case presentation**

 The anticoagulation therapy included warfarin with a target international normalized ratio of 2.5–3.5 and was associated with oral aspirin (100 mg/day) as the patient was a woman in fertile age living in a rural area.

**I notice that the author uses two specific mechanic valves in aortic and mitral position. On the market is available a different mechanical valve prosthesis, that can be used in both mitral and aortic position. For this specific mechanical valve the anticoagulation therapy can be carried out with a lower INR in combination with aspirin.  Has the author thought to change the used mechanical valves in consideration to the special social contest in which he practices and the difficulties for the patients to adhere to the needed anticoagulation therapy?**

 The Reviewer is correct and, indeed, the On-X valve was evaluated for possible use in our patients. Despite the possible better outcomes in our patients, the costs of this valve are higher compared to the valve which are currently used at the Salam Center. The Salam Center is financially supported by the local government and by an NGO called “Emergency”. Based on the limited financial resources available, unfortunately the routine use of the On-X valve was not possible and, thus, was limited to very few selected patients (i.e. redo patients).

**The author states that the patient had a low compliance with regard to the anticoagulation therapy, but no information is given if the patient had trombi on the mitral and aortic valves or if she had peripheral embolism.**

 We thank the Reviewer for this remark. We specified in the case presentation that the patient did not have any clot formations nor embolism events.

**Changes: case presentation**

 No other masses or possible thrombi were noticed on the left heart prosthetic valves, nor episodes of systemic or pulmonary embolism were reported.

**To remove the mass in the right atrium, the patient underwent a second surgery and the heart was arrested. Why the author used this surgical approach? This kind of surgery can be performed as well with a beating heart. Since the patient had a mildly depressed** **left ventricular systolic function (46%), why to expose this heart to ischaemic time when it was possible to avoid it?**

 We agree with the Reviewer on the fact that operations on the right heart could be completed with a beating heart approach. Indeed, this is the approach used at Salam Centre for most surgeries on tricuspid valves. However, in this case, a possible diagnosis of malignant tumour could not be ruled out before surgery. Therefore, the heart was stopped to reduce the risk of tumour embolization resulting from mass manipulation and allow for a radical removal of the mass in case of malignant diagnosis.

**Changes: case presentation**

 The surgery was completed at arrested heart to reduce the risk of neoplastic embolization in case of malignant diagnosis and allow for a radical removal of the mass.

**I think the the article can be deemed suitable for indexing with minor corrections.**

 We thank the Reviewer for the attention and efforts in reviewing our manuscript.

# Peer review: Reviewer response for version 1

**DOI:** 10.5256/f1000research.141821.r158878

**Published:** 2023-03-08

**Authors:** Tong Li

**Affiliations:** 1 Thoracic Research Centre, Collegium Medicum Nicolaus Copernicus University, Bydgoszcz, Poland

The authors contributed a very rare and meaningful case to us. The etiological background, preoperaitve exams and surgery details have been clearly presented in this case report. There are only several known cases of right atrial thrombus after tricuspid annuloplasty published in the history. The known cases are all old patients from east Asia, which the patients also maybe experienced the country developing processes. From this young African girl's stroy, the doctors in the developing countries maybe reinforce their postoperaive and follow-up guidances to the patients, especially those who live in rural areas. Meanwhile, the surgeon can be more focused on and cautious to the intraoperative procedures to facilitate the long term results, for example the De Vega annuloplasty, considering their patients may not have statifying compliance. I would like to suggest the authors adding the patients' BMI at 13 years old and 16 years old to let the whole story more vivid.

Are enough details provided of any physical examination and diagnostic tests, treatment given and outcomes?

Yes

Is the case presented with sufficient detail to be useful for other practitioners?

Yes

Is sufficient discussion included of the importance of the findings and their relevance to future understanding of disease processes, diagnosis or treatment?

Yes

Is the background of the case’s history and progression described in sufficient detail?

Yes

Reviewer Expertise:

mechanical circulatory support and heart transplantation

I confirm that I have read this submission and believe that I have an appropriate level of expertise to confirm that it is of an acceptable scientific standard.

## References

[1] : [Pedunculated and Mobile Right Atrial Thrombus Moderately after Mitral Valve Plasty and Tricuspid Valve Annuloplasty:Report of a Case]. *Kyobu Geka* .2022;75(4) :312-315 35342164

# Response

**Published:** 2023-05-30

**Authors:** Salvatore Lentini

**Affiliations:** The Salam Centre for Cardiac Surgery, Sudan

We are grateful for the opportunity to submit a revised version of our manuscript ******“****** ******Case Report: Right atrial organized thrombus three years after tricuspid annuloplasty****** ******”****** to *F1000 Research* and are thankful for the very constructive and helpful comments of the Reviewers. In addition to responding to the comments, we revised the manuscript, and hope that the changes and responses satisfy the Editors and Reviewers` requests.

****Reviewer 1****

**The authors contributed a very rare and meaningful case to us. The etiological background, preoperaitve exams and surgery details have been clearly presented in this case report. There are only several known cases of right atrial thrombus after tricuspid annuloplasty published in the history. The known cases are all old patients from east Asia, which the patients also maybe experienced the country developing processes. From this young African girl's story, the doctors in the developing countries maybe reinforce their postoperaive and follow-up guidances to the patients, especially those who live in rural areas. Meanwhile, the surgeon can be more focused on and cautious to the intraoperative procedures to facilitate the long term results, for example the De Vega annuloplasty, considering their patients may not have statifying compliance. I would like to suggest the authors adding the patients' BMI at 13 years old and 16 years old to let the whole story more vivid.**

 We thank the reviewer for his positive evaluation on our manuscript. As suggested, we added the patient’s BMI to the case report description.

**Changes: case presentation**

 She was first referred to our hospital due to severe mitral, aortic and tricuspid regurgitation when she was 13 years old (body mass index 12.65).

 […]

 At the follow-up visit 41 months after surgery (16 years old, body mass index 18.49)
